# Cell Chromatography-Based Screening of the Active Components in Buyang Huanwu Decoction Promoting Axonal Regeneration

**DOI:** 10.1155/2019/6970198

**Published:** 2019-09-29

**Authors:** Xiangli Yan, Shengxin Wang, Aiming Yu, Xiao Shen, Haozhen Zheng, Lisheng Wang

**Affiliations:** College of Chinese Materia Medica, Guangzhou University of Chinese Medicine, Guangzhou 51006, Guangdong, China

## Abstract

Buyang Huanwu decoction (BHD), a popular formulation prescribed in traditional Chinese medicine (TCM) for the treatment of ischemic stroke, has been reported to have a potential role in promoting axonal regeneration. The purpose of the study was to screen and identify bioactive compounds from BHD using live PC12 cells coupled with high-performance liquid chromatography-tandem mass spectrometry (HPLC-MS/MS). Using this approach, we successfully identified six bioactive components from BHD. These components have protective effects on oxygen-glucose deprivation/reperfusion (OGD/R) injury to PC12 cells. Furthermore, calycosin-7-d-glucoside (CG) and formononetin-7-O-*β*-d-glucoside (FG) could upregulate the protein expression of growth-associated protein 43 (GAP-43) and brain-derived neurotrophic factor (BDNF). This study suggests that living cells combined with HPLC-MS/MS can be used for the screening of active ingredients in TCMs.

## 1. Introduction

Ischemic stroke is a brain disease marked by insufficient blood supply to brain tissues, with high morbidity and mortality. The limited degree of spontaneous recovery after stroke translates into profound economic and emotional burdens. Axonal injury blocks normal cell-to-cell interactions or neural circuits in the ischemic brain, which usually occurs before neuronal death due to energy depletion or cerebral edema [1]. It has been estimated that 7 miles of axons will be lost every minute after ischemic stroke [2]. This astonishing loss of the connections between neurons indicates the role of axons in poststroke repair [3]. However, the brain has some ability to compensate for the functions lost after a stroke owing to the remarkable plasticity of this organ [4]. A large body of evidence suggests that axonal regeneration plays a key role in the recovery of brain function because it can establish new neural connections to alleviate brain function loss caused by stroke [5–7].

Buyang Huanwu decoction (BHD) was created by Qingren Wang, comprising Radix Astragali, Semen Persicae, Radix Angelicae Sinensis, Flos Carthami, Rhizoma Ligustici Chuanxiong, Radix Paeoniae Rubra, and Lumbricus. In recent years, there have been more and more reports on this formulation owing to its significant neuroprotective properties. BHD administration following spinal cord injury has been reported to protect injured neurons, promote regeneration, and enhance functional recovery [8]. Other studies have shown that BHD can promote the axonal regeneration of injured sciatic nerves and transverse peripheral nerves [9, 10]. Although BHD has been demonstrated to boost axonal regeneration effects, the effective components of BHD exerting the neuroprotective effects still need to be investigated.

Modern studies have proven that for the optimum biological activity of a drug, it should interact with its targets on cell membranes [11, 12]. Therefore, cell membrane chromatography (CMC) has been employed as a promising approach for screening bioactive components binding specific receptors in complex systems [12–16]. However, due to the shedding of the cell membrane, this system suffers from the short life and poor reproducibility. These make their applications limited. Many researchers have begun to use nanotechnology to screen active ingredients from natural products. Hu et al. [17] have screened two bioactive components using magnetic carbon nanotubes camouflaged with cell membrane and pharmacologically verified that lappaconitine can fight *α*_1A_-AR; Ding et al. [18] have used APTES-decorated HepG2 cancer stem cell membrane chromatography to screen three bioactive ingredients. Cell proliferation and apoptosis experiments showed that they could inhibit HepG2 cancer stem cells. Nanotechnology improves efficiency and targeting, but the external environment in which cells survive cannot be maintained, so we screened active ingredients with living cells as the stationary phase. Briefly, sample solution incubates cells, and washing solution elutes unbound components; then cells are incubated with dissociation solution; the targets on the cell membrane are inactivated, collecting the dissociation liquid; and the target components are confirmed by HPLC-MS/MS. In the previous study, we successfully screened the angiogenesis-promoting ingredients in BHD by brain microvascular endothelial cells and screened the antithrombotic components by platelets [19, 20]. Accordingly, we attempted to screen the active ingredients that promote axonal regeneration in BHD by using PC12 cells. PC12 cells are derived from rat adrenal pheochromocytoma cells and have neuronal characteristics after induction. Therefore, PC12 cells are commonly used in the study of neurological diseases. The protective effect of potential active compounds on cell damage induced by oxygen-glucose deprivation/reperfusion (OGD/R) was determined by cell viability assay, and the promoted axonal regeneration activity was tested by examining the expression of growth-associated protein 43 (GAP-43) and brain-derived neurotrophic factor (BDNF) using western blotting.

## 2. Materials and Methods

### 2.1. Materials

Radix Astragali, Semen Persicae, Radix Angelicae Sinensis, Flos Carthami, Rhizoma Ligustici Chuanxiong, Radix Paeoniae Rubra, and Lumbricus were purchased from Guangzhou Zhixin Chinese Herbal Pieces Co., Ltd. (Guangzhou, China) and authenticated by Professor Wei Li of the Guangzhou University of Chinese Medicine. Standards of 6-hydroxykaempferol-tri-O-glucoside, 6-hydroxykaempferol-di-O-glucoside, calycosin-7-O-β-d-glucoside, galloyl-paeoniflorin, formononetin-7-O-*β*-d-glucoside, and (3R)-7,2′-hydroxy-3′,4′-dimethoxy-isoflavan were from Chengdu Keloma Biotechnology Co., Ltd. (Chengdu, China); the purity was greater than 98%. HPLC-grade acetonitrile and methanol were acquired from Merck (Darmstadt, Germany); HPLC-grade water was prepared using a Milli-Q system (Bedford, MA, USA). Fetal bovine serum (FBS) was obtained from PAN-Biotech (Aidenbach, Germany); horse serum (HS) and Dulbecco's modified Eagle's medium (DMEM) were from Sigma-Aldrich (St. Louis, MO, USA); nerve growth factor (NGF) was procured from R&D Systems (Minneapolis, MN, USA); VEGF was from Beijing Cheng Lin Biological Technology Co., Ltd (Beijing, China). Anti-GAP-43, anti-BDNF, and anti-*β*-tubulin (TUJ1) antibodies were supplied by Abcam (Cambridge, UK). Goat serum and goat anti-mouse IgG-FITC conjugate were purchased from ZSGB Biotechnology Co., Ltd. (Beijing, China); and sputum serum and sputum anti-sheep IgG-FITC conjugate were obtained from Jackson ImmunoResearch (West Grove, PA, USA). 4,6-Diamidino-2-phenylindole (DAPI) was from Beyotime Institute of Biotechnology (Shanghai, China); Cell Counting Kit-8 (CCK-8) was obtained from Dojindo Laboratories (Kumamoto, Japan).

### 2.2. Preparation of BHD

BHD (111 g) was composed of Radix Astragali, Semen Persicae, Radix Angelicae Sinensis, Flos Carthami, Rhizoma Ligustici Chuanxiong, Radix Paeoniae Rubra, and Lumbricus at a 60 : 9 : 9 : 9 : 6 : 9 : 9 ratio. The extraction and preparation process of BHD was according to our previous study [19]. In short, the herbs were soaked in water for 0.5 h, and then extracted twice in a water bath, concentrated under reduced pressure, and was purified using D101 macroporous resin to collect 40% and 70% ethanol eluent. The eluent was concentrated to a final concentration of 1.1 g/mL, filtered using a 0.22 *μ*m membrane, and detected by HPLC. The concentration was diluted to 110 mg/mL for screening of active ingredients in BHD.

### 2.3. Cultivation and Induced Differentiation of PC12 Cells

PC12 cells were resuspended in medium composed of DMEM, 10% HS, 5% FBS, 100 U/mL penicillin, and 100 mg/mL streptomycin; seeded on a tiny glass sheet at a density of 5 × 10^3^ cells/mL; and incubated in an incubator containing 5% CO_2_ at 37°C. After 12 h, induction of PC12 cells differentiation was initiated by medium containing 10 ng/ml nerve growth factor (NGF). The medium was altered every 2 days, and immunofluorescence was used to detect induced PC12 cells. Briefly, PC12 cells were washed three times with PBS and fixed with 4% paraformaldehyde for 30 min. The cell membranes were permeated with 0.2% Triton X-100 for 0.5 h, blocked in 10% goat serum for 1 h, and incubated with anti-TUJ1 (1 : 50) or anti-GAP-43 (1 : 50) antibodies overnight at 4°C. Then, the cells were incubated with goat anti-mouse IgG-FITC conjugate and anti-sheep IgG-FITC conjugate at 37°C for 1 h, and the nuclei were stained with DAPI for 5 min at 37°C. Pictures were obtained under a laser confocal microscope (Olympus).

### 2.4. Biospecific Live-Cell-Based Isolation of BHD Components

PC12 cells were seeded in 75 cm^2^ culture flasks, induced with NGF for 4 days, and digested with trypsin. Cell pellets were collected and incubated with 15 mL of BHD (110 mg/mL) for 1 h at 37°C; the blank group was incubated with the same volume of medium. The cell pellets were collected and washed with PBS (pH 7.4) five times to remove unbound components. The bound BHD materials were dissociated with PBS (pH 4.0) at 37°C for 1 h and centrifuged (500 ×*g*). The supernatant was collected and subjected to solid-phase extraction for purification and enrichment. The eluents were collected and dried under a gentle flow of nitrogen at 40°C. Each sample was made up to 1 mL and filtered through a 0.22 *μ*m membrane to be analyzed. The experiment was replicated thrice.

### 2.5. HPLC-MS/MS

An HPLC analysis system equipped with an SIL-82A autosampler, LC-20A pump, and SPD-20A detector was used, which was controlled by LC Solution software (Shimadzu, Japan). The column used was Hypersil ODS2 (4.6 × 250 mm, 5 *μ*m); mobile phase A was acetonitrile, and mobile phase B was 0.1% formic acid in water. The elution condition was as follows: 0–5 min, 4% A; 5–15 min, 4%–6% A; 15–20 min, 6%–8% A; 20–35 min, 8%–10% A; 35–70 min, 10–15% A; 70–105 min, 15%–20% A; 105–140 min, 20%–35% A; and 140–160 min, 35%–60% A. Detection wavelength was 250 nm, column temperature was 30°C, and flow rate was 1.0 mL/min.

The potential active components dissociated from PC12 cells were analyzed by the AB SCIEX TripleTOF 5600^+^ System (Framingham, MA, USA). The optimal operating parameters were as follows: ion spray voltage floating, 4500 V; ion source gas 2, 55 psi; ion source gas 1 (N_2_) flow, 55 psi; curtain gas, 35 psi; temperature, 500°C; and scan range, 100–1000 Da.

### 2.6. OGD/R Model

We inoculated PC12 cells into poly-l-lysine-coated 96-well plates at a density of 5 × 10^4^ cells/mL. Cells were induced to differentiate by NGF. When the cell density reached 90%–95%, we removed the medium, washed the cells thrice with PBS, and incubated them in a three-gas incubator (94% N_2_, 5% CO_2_, and 1% O_2_) with glucose-free medium for 6 h at 37°C to mimic hypoxic injury (OGD). Next, the medium was replaced with complete medium and incubated in an incubator with 5% CO_2_ at 37°C for 24 h.

### 2.7. Drug Treatment

To determine the effect of combined components on cell viability after OGD/R injury, PC12 cells were divided into three groups: control (PC12 cells without OGD/R); model (PC12 cells with OGD/R); drug intervention (glucose-free medium in the reoxygenation stage was replaced with complete medium containing different concentrations of binding components). Standard compounds were dissolved in DMSO and diluted in complete medium to the appropriate concentrations: 6-hydroxykaempferol-tri-O-glucoside at 1.130, 5.650, and 11.300 *μ*g/mL; 6-hydroxykaempferol-di-O-glucoside at 1.365, 6.825, and 13.650 *μ*g/mL; calycosin-7-O-*β*-d-glucoside at 1.063, 5.313, and 15.938 *μ*g/mL; galloyl-paeoniflorin at 2.240, 11.200, and 22.400 *μ*g/mL; formononetin-7-O-*β*-d-glucoside at 0.163, 1.630, and 16.300 *μ*g/mL; and (3R)-7,2′-hydroxy-3′,4′-dimethoxyisoflavan at 0.800, 4.000, and 12.000 *μ*g/mL.

To investigate the effect of combined components on axon growth after OGD/R injury, PC12 cells were divided into five groups: control (PC12 cells without OGD/R); model (PC12 cells with OGD/R); calycosin-7-O-*β*-d-glucoside (glucose-free medium in the reoxygenation stage was replaced with complete medium containing 16 *μ*g/mL CG for 24 h); formononetin-7-O-*β*-d-glucoside (glucose-free medium in the reoxygenation stage was replaced with complete medium containing 12 *μ*g/mL FG for 24 h); VEGF (glucose-free medium in the reoxygenation stage was replaced with complete medium containing 10 ng/mL VEGF for 24 h).

### 2.8. Cell Viability Assay

The effects of various components on the viability of PC12 cells after OGD/R were assessed using CCK-8 kit. After 24 h of treatment, CCK-8 was added to the medium for another 2 h at 37°C. Then, we measured the absorbance (OD) at 450 nm using a microplate reader.

### 2.9. Western Blot

After exposure to OGD/R, total protein was extracted after the cells were lysed by RIPA, and the protein concentration was determined using BCA protein assay kit. The lysates were boiled at 100°C for 10 min. Ten *μ*g of total protein samples were loaded onto 10% SDS-PAGE gel and transferred to PVDF membranes. After the membranes were blocked with 5% nonfat milk, they were incubated with primary antibodies against GAP-43 (1 : 1000 dilution) and BDNF (1 : 1000 dilution) overnight at 4°C. Then, the membrane was incubated with secondary antibodies for 1 h at room temperature. Finally, the membranes were exposed using the Bio-Rad ChemiDoc Touch Imaging System and analyzed with Image J software. The relative protein levels were normalized to *β*-actin.

### 2.10. Statistical Analysis

The results were expressed as mean ± standard deviation and analyzed by the statistical software SPSS 20.0 software. *P* ≤ 0.05 was considered statistically significant.

## 3. Results

### 3.1. Preparation of BHD

Radix Astragali constitutes a major part of BHD. Samples prepared by the water extraction and alcohol precipitation method in a previous study were too viscous, which was not conducive to the development of cell membrane solid-phase chromatography. Therefore, a macroporous resin column was used to remove polysaccharides and collect effective constituents such as glycosides. HPLC fingerprinting experiments were carried out according to Liao et al. [19]. Results showed that the similarity between the prepared BHD and control was over 0.9, indicating that the sample met the requirements and could be used in subsequent experiments.

### 3.2. Cultivation and Induced Differentiation of PC12 Cells

**Figure 1:**
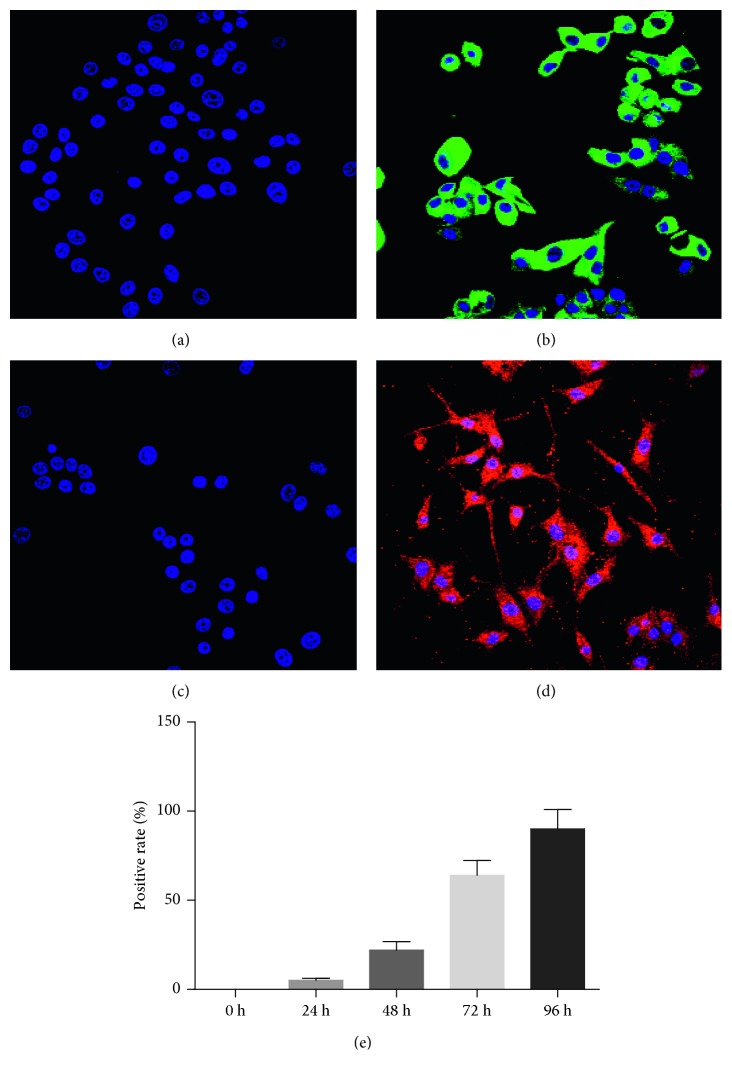
Effects of NGF on PC12 cells. (a) The expression of TUJ1 protein was induced by NGF on the zeroth day. (b) The expression of TUJ1 protein was induced by NGF on the fourth day. (c) The expression of GAP-43 protein was induced by NGF on the zeroth day. (d) The expression of GAP-43 2 protein was induced by NGF on the fourth day. (e) Positive rate of cells after different induction durations. Data are expressed as mean ± S. D. of independent experiments.

Undifferentiated PC12 cells do not possess neuronal characteristics; hence, determining the optimal time to induce differentiation is a critical step. Here, we used NGF-induced cell differentiation and examined the effects of different induction durations on PC12 cells. The results of laser confocal microscopy showed that by NGF induction, PC12 cells expressed neuronal characteristic proteins TUJ1 and GAP-43 on the fourth day (Figure 1).

### 3.3. Biospecific Live-Cell-Based Isolation and HPLC-MS/MS Identification of BHD Components

**Figure 2:**
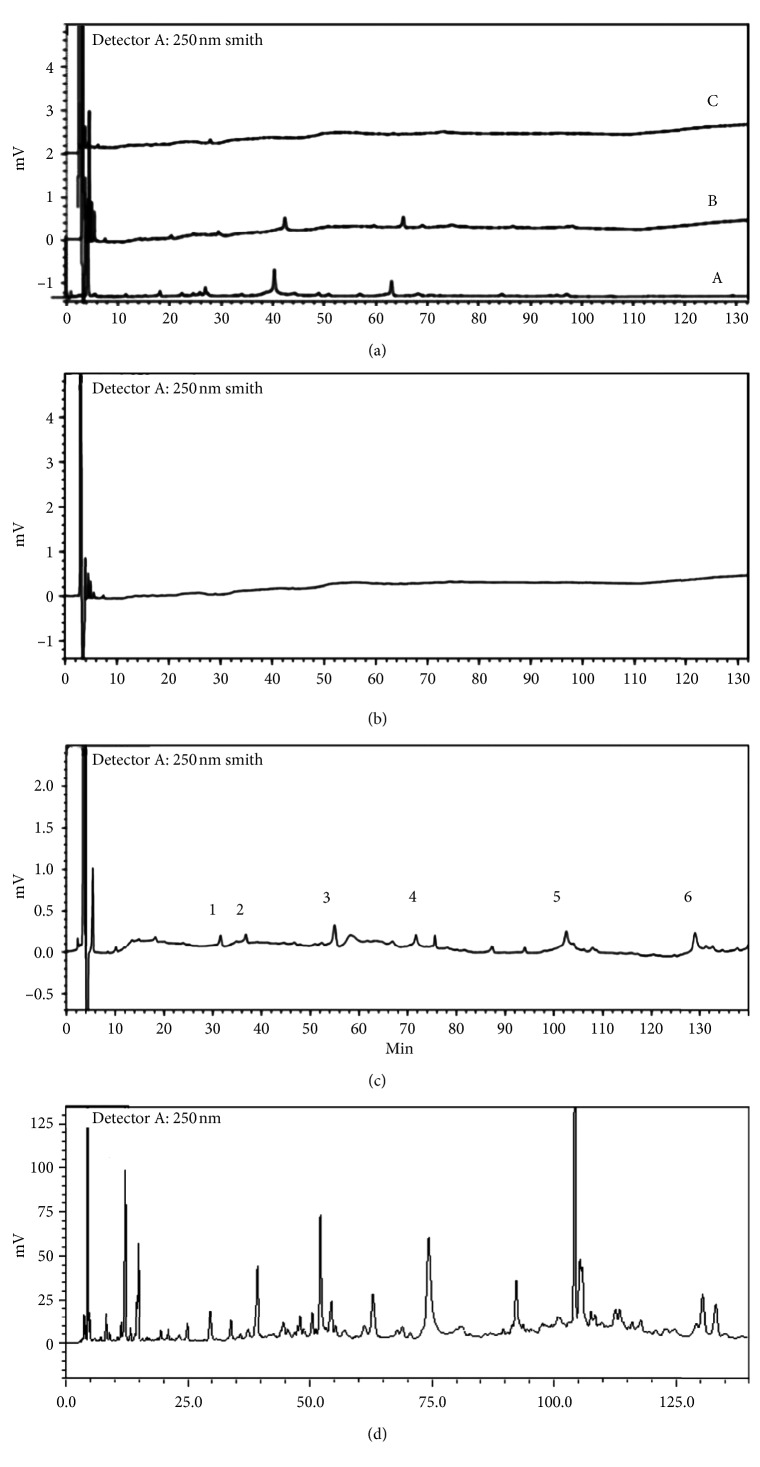
Chromatograms of washing solution, dissolution solution, and total extracts of BHD: (a) washing solution (A) the first washing solution, (B) the second washing solution, (C) the third washing solution; (b) blank group dissolution solution; (c) BHD incubation group dissociation solution; (d) total extracts of BHD.

**Figure 3:**
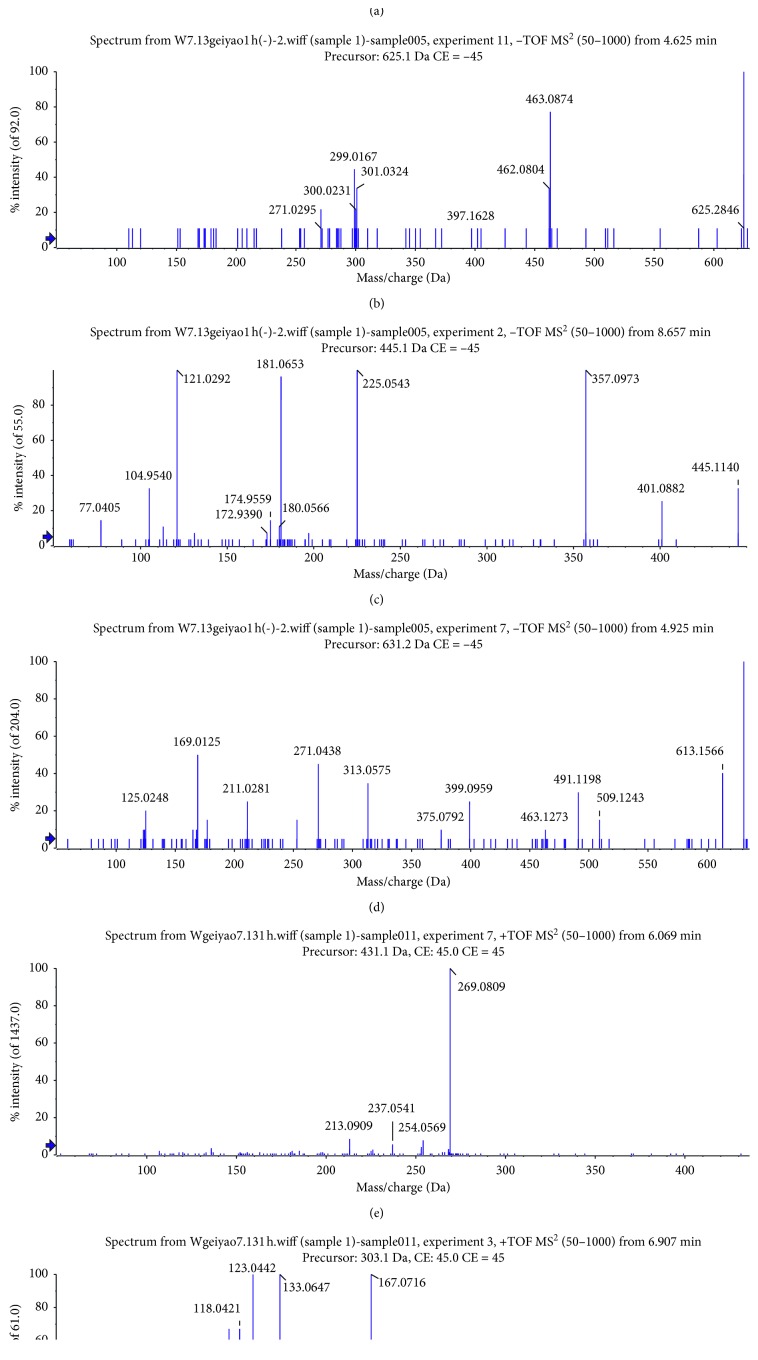
MS spectra of the six binding components. (a) 6-Hydroxykaempferol-tri-O-glucoside; (b) 6-hydroxykaempferol-di-O-glucoside; (c) calycosin-7-O-*β*-d-glucoside; (d) galloyl-paeoniflorin; (e) formononetin-7-O-*β*-d-glucoside; (f) (3R)-7,2′-hydroxy-3′,4′-dimethoxyisoflavan.

**Figure 4:**
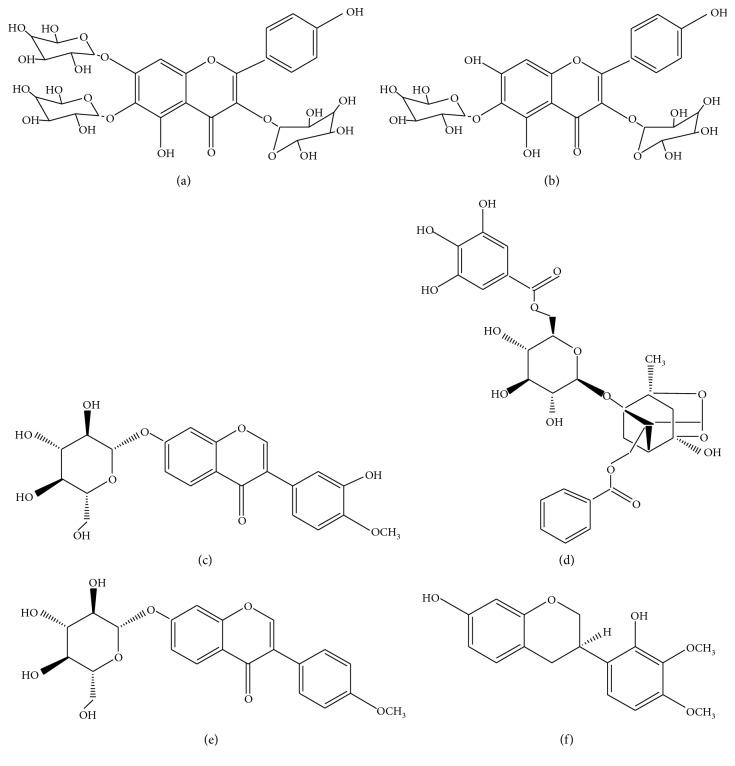
The structures of the identified binding components. (a) 6-Hydroxykaempferol-tri-O-glucoside; (b) 6-hydroxykaempferol-di-O-glucoside; (c) calycosin-7-O-*β*-D-glucoside; (d) galloyl-paeoniflorin; (e) formononetin-7-O-*β*-D-glucoside; (f) (3R)-7,2′-hydroxy-3′,4′-dimethoxyisoflavan.

To ensure that the unbound components were completely washed, the cell pellets were washed with PBS three times. As shown in Figure 2(a), no peaks were detected by HPLC in the washing solution of the last washing solution of PC12 cells incubated with BHD. At the same time, six peaks were detected (Figure 2(c)) and identified by comparison with the mass spectra reported in the literature [21] (Figure 3). These peaks represented 6-hydroxykaempferol-tri-O-glucoside, 6-hydroxykaempferol-di-O-glucoside, calycosin-7-O-*β*-d-glucoside, galloyl-paeoniflorin, formononetin-7-O-*β*-d-glucoside, and (3R)-7,2′-hydroxy-3′,4′-dimethoxyisoflavan (Figure 4).

### 3.4. Cell Viability Assay

**Figure 5:**
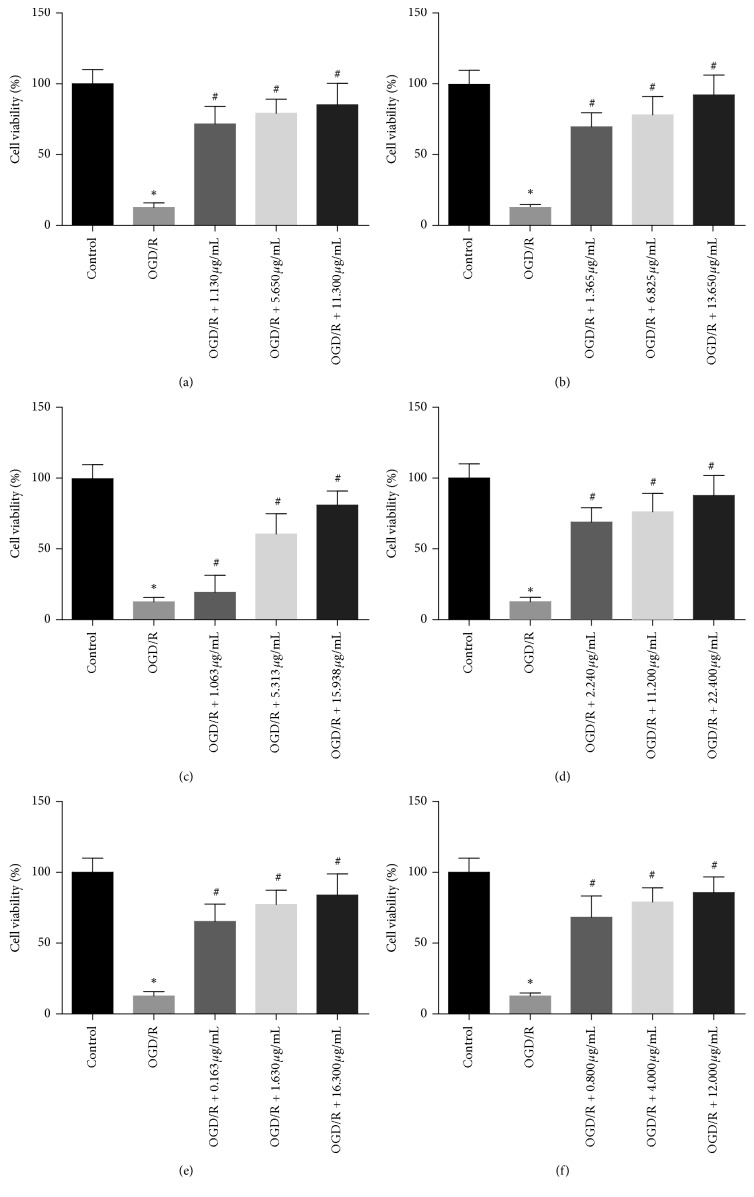
Protective effects of binding components on PC12 cells. (a) 6-Hydroxykaempferol-tri-O-glucoside; (b) 6-hydroxykaempferol-di-O-glucoside; (c) calycosin-7-O-*β*-d-glucoside; (d) galloyl-paeoniflorin; (e) formononetin-7-O-*β*-d-glucoside; (f) (3R)-7,2′-hydroxy-3′,4′-dimethoxyisoflavan. ^*∗*^*P* < 0.05*vs.* control group; ^#^*P* < 0.05*vs.* OGD/R group.

To explore the protective effects of binding components on PC12 cells with OGD/R injury, we performed cell viability assay. As shown in Figure 5, after the cells were treated with OGD/R, the viability was significantly lower than that of the normal cells (*P* < 0.05), indicating that the OGD/R model was successfully established. However, the viability of cells after OGD/R can be significantly improved by 6-hydroxykaempferol-tri-O-glucoside (1.130–11.300 *μ*g/mL), 6-hydroxykaempferol-di-O-glucoside (1.365–13.650 *μ*g/mL), calycosin-7-O-*β*-d-glucoside (1.063–15.938 *μ*g/mL), galloyl-paeoniflorin (2.240–22.400 *μ*g/mL), formononetin-7-O-*β*-d-glucoside (0.163–16.300 *μ*g/mL), and (3R)-7,2′-hydroxy-3′,4′-dimethoxy-isoflavan (0.800–12.000 *μ*g/mL) (*P* < 0.05), and they all showed dose dependency.

### 3.5. Expression of GAP-43 and BDNF

**Figure 6:**
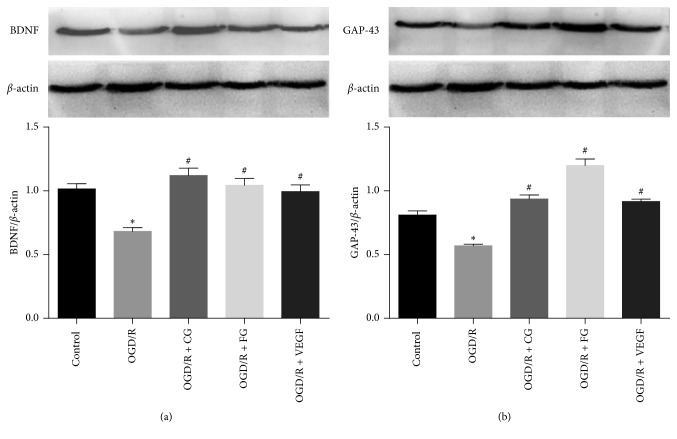
Effects of CG and FG on protein expression in PC12 cells. ^*∗*^*P* < 0.05*vs.* control group; ^#^*P* < 0.05*vs.* OGD/R group.

The results of CCK-8 test showed that all the selected compounds had protective effects on cell damage caused by OGD/R. Among the identified components, we investigated the protective effects of calycosin-7-O-*β*-d-glucoside and formononetin-7-O-*β*-d-glucoside on PC12 cells with injury by detecting the expression of GAP-43 and BDNF proteins. As shown in Figure 6, the expression of GAP-43 and BDNF were downregulated after OGD/R compared to the control group. The expression of GAP-43 and BDNF proteins was significantly increased after the intervention of calycosin-7-O-*β*-d-glucoside and formononetin-7-O-*β*-d-glucoside (*P* < 0.05).

## 4. Discussion

Traditional Chinese medicine is an unknown complex system. It is quite necessary to explore the basis of its pharmacodynamics. The traditional extraction and separation methods have a long cycle and a low hit rate. Cell membrane chromatography uses the principle of combining drugs with targets on cell membranes to achieve screening of active pharmaceutical ingredients. The method uses living cells as a stationary phase, the integrity of the cell membrane and the surrounding environment is maintained, and the interference of nonactive substances is eliminated, which is a rapid and efficient screening method. In this study, six components were identified by comparison with the MS spectra reported in the literature, 6-hydroxykaempferol-tri-O-glucoside, 6-hydroxykaempferol-di-O-glucoside, calycosin-7-O-*β*-d-glucoside, galloyl-paeoniflorin, formononetin-7-O-*β*-d-glucoside, and (3R)-7,2′-hydroxy-3′,4′-dimethoxyisoflavan.

Calycosin-7-O-*β*-d-glucoside is a representative isoflavone in Radix Astragali, the principal drug component of BHD, and it has been widely used for the treatment of many diseases [22]. A previous study revealed that calycosin-7-O-*β*-d-glucoside possesses cytoprotective effects in l-glutamate-treated PC12 cells [23] and might protect blood-brain barrier integrity by modulating the NO/cav-1/MMP pathway [24]. Another study showed that calycosin-7-O-*β*-d-glucoside could alleviate ischemia-reperfusion injury through the PI3K/Akt pathway [25]. Galloyl-paeoniflorin can inhibit oxidative stress and reduce neuroinflammation and cell damage after cerebral ischemia-reperfusion and apoptosis of the mitochondrial pathway [26, 27]. The efficacy of other ingredients in OGD/R-treated neuron cells has not been reported. This study reports for the first time the effects of novel ingredients on the viability of PC12 cells.

GAP-43 is a neuron-specific axonal membrane protein, and it is a marker of axonal regeneration [28, 29]. BDNF is a neurotrophic factor widely distributed in the central and peripheral nervous system. It plays important roles in promoting cell differentiation, nerve growth, and neuronal survival, as well as in maintaining normal brain function [30]. Among the identified components, we investigated the protective effects of calycosin-7-O-*β*-d-glucoside and formononetin-7-O-*β*-d-glucoside on PC12 cells with injury by detecting the expression of GAP-43 and BDNF proteins. The results showed that calycosin-7-O-*β*-d-glucoside and formononetin-7-O-*β*-d-glucoside could promote axonal regeneration by upregulating the expression of GAP-43 and BDNF proteins.

## 5. Conclusion

In this study, we used a cell membrane chromatography approach to screen six active ingredients from BHD. They were 6-hydroxykaempferol-tri-O-glucoside, 6-hydroxykaempferol-di-O-glucoside, calycosin-7-O-*β*-d-glucoside, galloyl-paeoniflorin, formononetin-7-O-*β*-d-glucoside, and (3R)-7,2′-hydroxy-3′,4′-dimethoxyisoflavan. The ingredients were recognized to enhance cell activity in a dose-dependent manner. Calycosin-7-O-*β*-d-glucoside and formononetin-7-O-*β*-d-glucoside were deemed to promote the expression of GAP-43 and BDNF proteins.

However, among the identified compounds, we only explored the effects of calycosin-7-O-*β*-d-glucoside and formononetin-7-O-*β*-d-glucoside on axonal regeneration in PC12 cells. Next, we will study the effects of the remaining components on axonal regeneration in PC12 cells.

## Data Availability

The data are uploaded to 4TU. Centre for Research Data (the DOI of the dataset will be https://doi.org/10.4121/uuid:14c8d0f2-0a16-473f-9d8b-6e77969e76b6).

## Abbreviations

BHD: Buyang Huanwu decoction
CMC: Cell membrane chromatography
CCK-8: Cell Counting Kit-8
CG: Calycosin-7-d-glucoside
FG: Formononetin-7-O-*β*-d-glucoside
VEGF: Vascular endothelial growth factor
OGD/R: Oxygen-glucose deprivation/reperfusion
TCM: Traditional Chinese medicine.

## References

[1] Carmichael S. T., Kathirvelu B., Schweppe C. A., Nie E. H. (2017). Molecular, cellular and functional events in axonal sprouting after stroke. *Experimental Neurology*.

[2] Saver J. L., Cushman M. (2018). Striving for ideal cardiovascular and brain health: it is never too early or too late. *JAMA*.

[3] Hinman J. D. (2014). The back and forth of axonal injury and repair after stroke. *Current Opinion in Neurology*.

[4] Sun Y., Cheng X., Wang H. (2017). DL-3-*n*-butylphthalide promotes neuroplasticity and motor recovery in stroke rats. *Behavioural Brain Research*.

[5] Murphy T. H., Corbett D. (2009). Plasticity during stroke recovery: from synapse to behaviour. *Nature Reviews Neuroscience*.

[6] Liu L., Yuan H., Yi Y. (2018). Ras-related C3 botulinum toxin substrate 1 promotes axonal regeneration after stroke in mice. *Translational Stroke Research*.

[7] Xiong X. X., Pan F., Chen R. Q. (2018). Neuroglobin boosts axon regeneration during ischemic reperfusion via p38 binding and activation depending on oxygen signal. *Cell Death & Disease*.

[8] Chen A., Wang H., Zhang J. (2008). BYHWD rescues axotomized neurons and promotes functional recovery after spinal cord injury in rats. *Journal of Ethnopharmacology*.

[9] Chang I. A., Lim H. D., Kim K. J., Shin H., Namgung U. K. (2016). Enhanced axonal regeneration of the injured sciatic nerve by administration of Buyang Huanwu decoction. *Journal of Ethnopharmacology*.

[10] Kim K.-J., Namgung U. (2018). Facilitating effects of Buyang Huanwu decoction on axonal regeneration after peripheral nerve transection. *Journal of Ethnopharmacology*.

[11] Han S., Zhang T., Feng L., Lv N., Wang S. (2013). Screening of target compounds from *Fructus Piperis* using high *α*1A adrenoreceptor expression cell membrane chromatography online coupled with high performance liquid chromatography tandem mass spectrometry. *Journal of Pharmaceutical and Biomedical Analysis*.

[12] Han S., Zhang P., Wei F., Wang S. (2012). Screening active components acting on *α*1A adrenergic receptors from agrimony using a Sprague-Dawley rat prostate cell membrane chromatography online coupled HPLC/MS method. *Analytical Methods*.

[13] Shi Y., He L., Wang S. (2006). Determination of ligustilide in rat blood and tissues by capillary gas chromatography/mass spectrometry. *Biomedical Chromatography*.

[14] Chen Y., Zhang N., Ma J. (2016). A platelet/CMC coupled with offline UPLC-QTOF-MS/MS for screening antiplatelet activity components from aqueous extract of Danshen. *Journal of Pharmaceutical and Biomedical Analysis*.

[15] Cao Y., Wang S., Li Y. (2018). A method for screening active components from Chinese herbs by cell membrane chromatography-offline-high performance liquid chromatography/mass spectrometry and an online statistical tool for data processing. *Journal of Chromatography A*.

[16] Fu J., Lv Y., Jia Q., Lin Y., Han S. (2019). Dual-mixed/CMC model for screening target components from traditional Chinese medicines simultaneously acting on EGFR & FGFR4 receptors. *Talanta*.

[17] Hu Q., Bu Y., Zhen X. (2019). Magnetic carbon nanotubes camouflaged with cell membrane as a drug discovery platform for selective extraction of bioactive compounds from natural products. *Chemical Engineering Journal*.

[18] Ding X., Cao Y., Yuan Y. (2016). Development of APTES-decorated HepG2 cancer stem cell membrane chromatography for screening active components from *Salvia miltiorrhiza*. *Analytical Chemistry*.

[19] Liao F., Meng Y., Zheng H. (2018). Biospecific isolation and characterization of angiogenesis-promoting ingredients in Buyang Huanwu decoction using affinity chromatography on rat brain microvascular endothelial cells combined with solid-phase extraction, and HPLC-MS/MS. *Talanta*.

[20] Liao F., Yu A., Yu J. (2018). Identification of active ingredients mediating anti-platelet aggregation effects of Buyang Huanwu decoction using a platelet binding assay, solid phase extraction, and HPLC-MS/MS. *Journal of Chromatography B*.

[21] Liu E.-H., Qi L.-W., Peng Y.-B. (2009). Rapid separation and identification of 54 major constituents in Buyang Huanwu decoction by ultra-fast HPLC system coupled with DAD-TOF/MS. *Biomedical Chromatography*.

[22] Li X., Qu L., Dong Y. (2014). A review of recent research progress on the astragalus genus. *Molecules*.

[23] Yu D., Duan Y., Bao Y., Wei C., An L. (2005). Isoflavonoids from *Astragalus mongholicus* protect PC12 cells from toxicity induced by L-glutamate. *Journal of Ethnopharmacology*.

[24] Fu S., Gu Y., Jiang J.-Q. (2014). Calycosin-7-O-*β*-d-glucoside regulates nitric oxide/caveolin-1/matrix metalloproteinases pathway and protects blood-brain barrier integrity in experimental cerebral ischemia-reperfusion injury. *Journal of Ethnopharmacology*.

[25] Ren M., Wang X., Du G., Tian J., Liu Y. (2016). Calycosin-7-O-*β*-d-glucoside attenuates ischemia-reperfusion injury in vivo via activation of the PI3K/Akt pathway. *Molecular Medicine Reports*.

[26] Yao C. W., Piao M. J., Kim K. C., Zheng J., Cha J. W., Hyun J. W. (2013). 6′-O-galloylpaeoniflorin protects human keratinocytes against oxidative stress-induced cell damage. *Biomolecules and Therapeutics*.

[27] Yao C. W., Piao M. J., Kim K. C. (2014). Cytoprotective effects of 6′-O-galloylpaeoniflorin against ultraviolet B radiation-induced cell damage in human keratinocytes. *In Vitro Cellular & Developmental Biology-Animal*.

[28] Schaden H., Stuermer C. A. O., Bähr M. (1994). Gap-43 immunoreactivity and axon regeneration in retinal ganglion cells of the rat. *Journal of Neurobiology*.

[29] Honer W. G., Falkai P., Chen C., Arango V., Mann J. J., Dwork A. J. (1999). Synaptic and plasticity-associated proteins in anterior frontal cortex in severe mental illness. *Neuroscience*.

[30] Shi N., Zhu C., Li L. (2016). Rehabilitation training and resveratrol improve the recovery of neurological and motor function in rats after cerebral ischemic injury through the Sirt1 signaling pathway. *BioMed Research International*.

